# Metal Bezoar-Induced Gastrointestinal Obstruction: A Rare Clinical Presentation

**DOI:** 10.7759/cureus.93873

**Published:** 2025-10-05

**Authors:** Edgar Alexis Flores Garcia, Axell Daniel Lugo Rodríguez, Emiliano De La Torre Pacheco, Jennifer Navarro Morales, José Ivan Rodríguez Murua, Jennifer Hermosillo Venegas, Jorge Alejandro Favela Ramos

**Affiliations:** 1 General Surgery, Hospital Nuevo Gómez Palacio, Gómez Palacio, MEX; 2 General Surgery, Unidad Médica de Alta Especialidad No. 71, Instituto Mexicano del Seguro Social, Torreón, MEX; 3 Legal Medicine, Fiscalía General de Justicia de Zacatecas, Zacatecas, MEX; 4 Internal Medicine, Hospital Nuevo Gómez Palacio, Gómez Palacio, MEX

**Keywords:** bezoar, endoscopic management, foreign body ingestion, gastrointestinal bezoar, gastrointestinal obstruction, metal bezoar, non-food bezoar, surgical removal, trichobezoar

## Abstract

Bezoars are accumulations of indigestible or non-food material within the gastrointestinal tract, capable of causing obstruction, bleeding, or perforation. We report the case of a 25-year-old male patient with a history of chronic polysubstance abuse who presented with a one-month history of persistent vomiting, severe epigastric pain, and minimal tolerance to liquids. Abdominal radiography and computed tomography revealed metallic foreign bodies in the stomach and colon. Endoscopic removal was deemed unfeasible due to the size and characteristics of the objects; therefore, open surgery was performed with successful extraction. Metal bezoars, as in this case, are rare and carry a high risk of complications, particularly gastrointestinal perforation, due to the ingestion of sharp or bulky objects. Treatment should be individualized based on bezoar type, size, and location. Endoscopic removal is the first-line approach when feasible; however, surgical intervention is warranted in complex or high-risk cases. This case underscores the need for a multidisciplinary approach combining surgical management, complication prevention, and treatment of underlying psychiatric or substance use disorders to reduce recurrence and improve long-term outcomes.

## Introduction

Bezoars have been described throughout history, dating back to the 18th century when they were believed to neutralize poison - hence their name. They were also historically used as treatments for epilepsy, infectious diseases, and as amulets to ward off evil spirits. The first trichobezoar was described in 1779 by Baudamant, and the first phytobezoar was reported by Quain in 1854, primarily composed of fruit remnants, notably coconut [1].

Bezoars are conglomerates of indigestible or non-food material that accumulate within the gastrointestinal tract, potentially causing obstruction, bleeding, or perforation. Phytobezoars, formed from indigestible plant material, are the most common type, accounting for approximately 70% of cases [1]. Less frequent types result from ingestion of unusual substances, including metal, plastic, toilet paper, or parasites such as *Ascaris lumbricoides *[2].

Bezoar formation is often associated with underlying medical or psychiatric conditions, as well as compulsive behaviors. Schizophrenia is reported in about 10% of bezoar cases, and trichotillomania - a disorder characterized by recurrent hair-pulling - has a global prevalence of 1% to 3% [3,4]. Ingestion of indigestible materials in these patients can lead to significant gastrointestinal complications requiring prompt diagnosis and intervention.

This report presents a rare case of a metal bezoar in a young adult with a history of polysubstance abuse, highlighting the diagnostic approach, surgical management, and the importance of a multidisciplinary strategy in addressing both the acute presentation and the underlying behavioral health factors.

## Case presentation

A 25-year-old male patient with no history of chronic degenerative diseases, but with a history of alcohol use and long-term polysubstance abuse (including methamphetamines, marijuana, and cocaine since early adolescence), was referred to the General Surgery service for suspected intestinal obstruction. The patient had a known psychiatric history, having been diagnosed with schizophrenia two years prior, but was reportedly unable to afford ongoing treatment. He reported a one-month history of persistent, non-bilious vomiting up to 10 times daily, accompanied by severe epigastric pain during each episode. He maintained his appetite but could tolerate only minimal liquids and no solid food.

**Figure 1:**
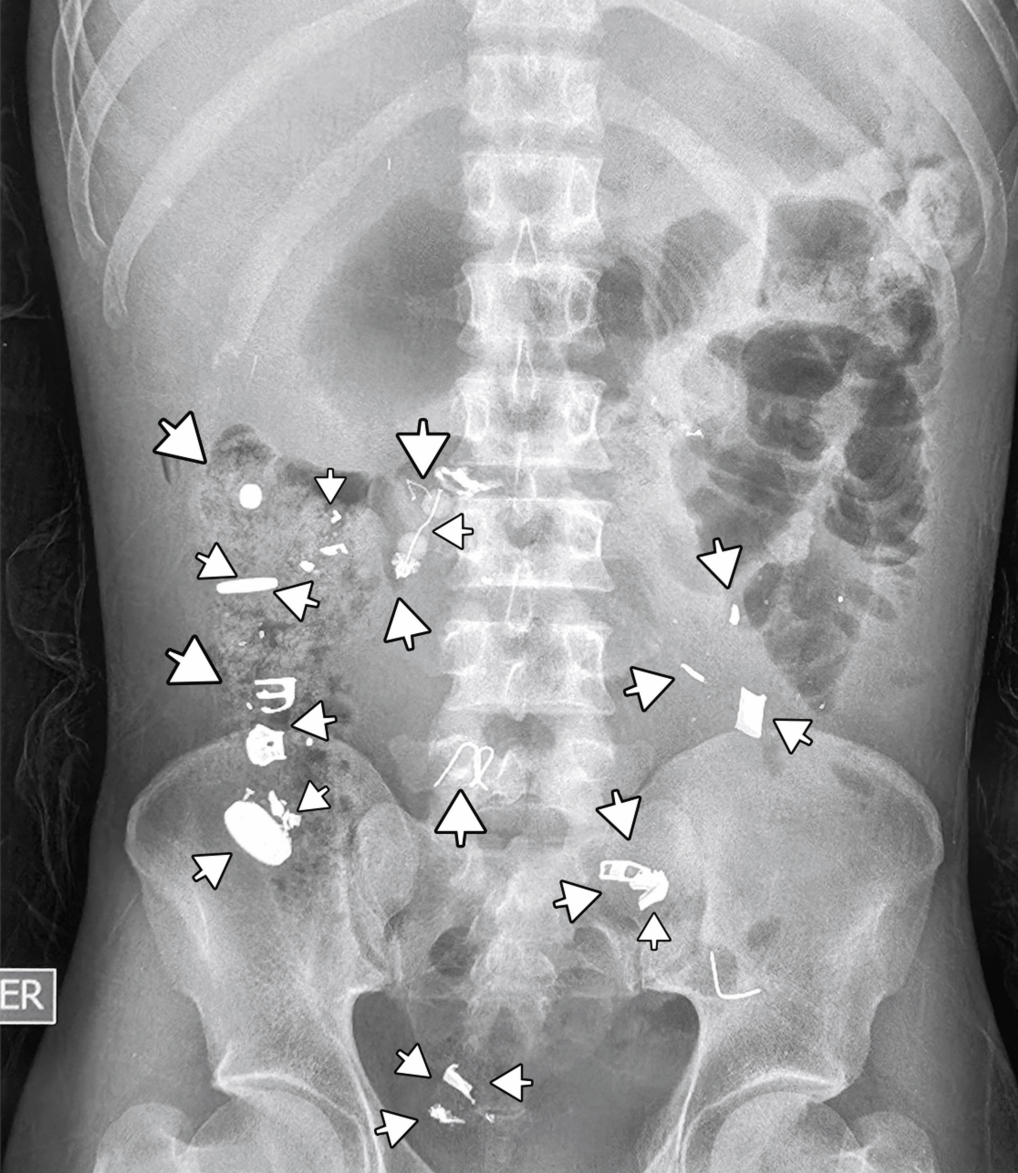
Plain abdominal X-ray showing metallic foreign bodies (arrows) in the colon.

**Figure 2:**
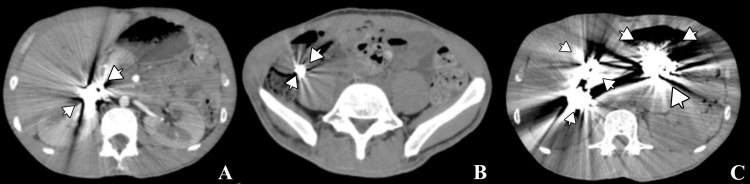
Abdominal computed tomography scan showing metallic foreign bodies (arrows) in the gastric chamber, small intestine, and colon. (A) Metallic foreign bodies in the duodenum. (B) Metallic foreign bodies in the colon. (C) Metallic foreign bodies in the gastric and duodenal chambers.

**Figure 3:**
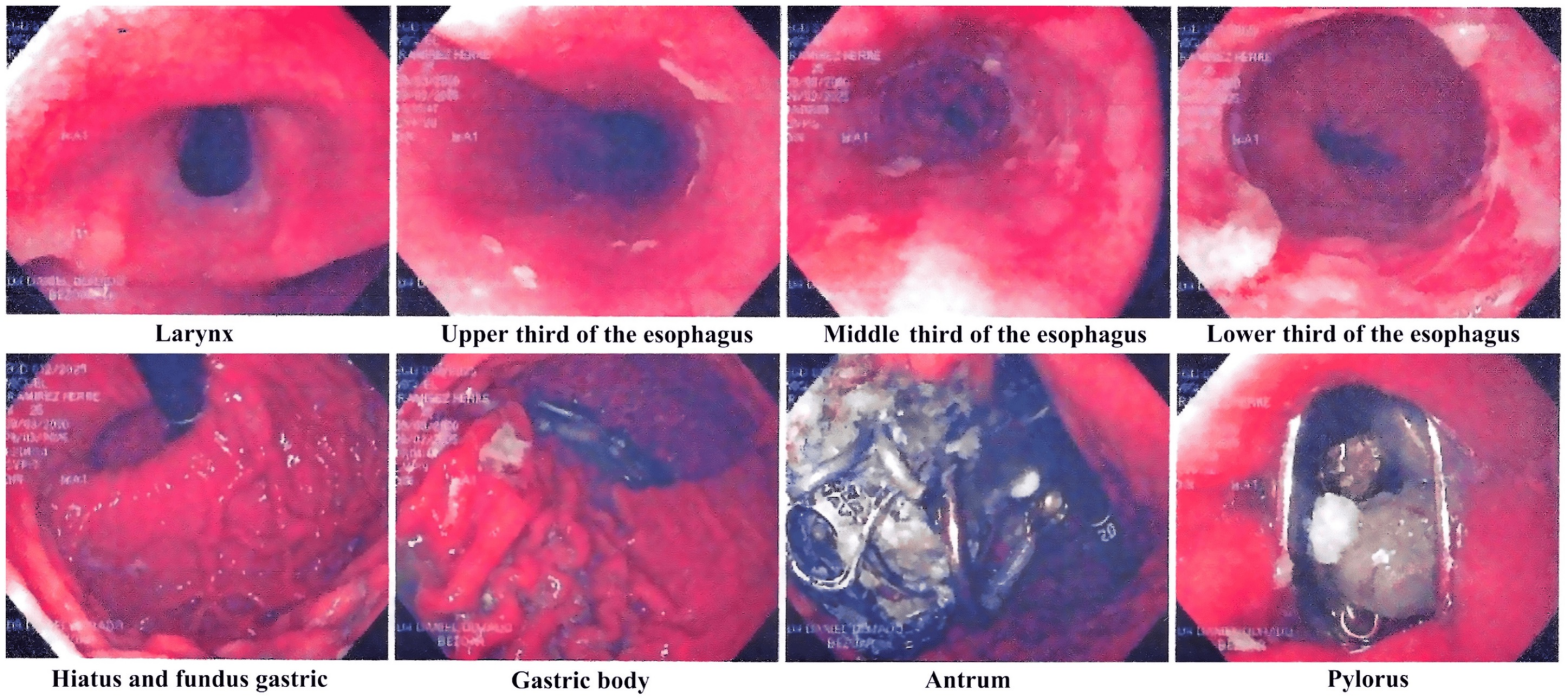
Diagnostic-therapeutic endoscopy in different portions of the gastrointestinal tract with the presence of metallic foreign bodies.

Imaging studies revealed radiopaque material within the colon on plain abdominal radiography (Figure 1) [5]. Contrast-enhanced computed tomography demonstrated metallic foreign bodies in the stomach and colon, although small intestine involvement could not be definitively excluded (Figure 2) [6]. Upper endoscopy confirmed metallic objects in the stomach; however, endoscopic extraction was not feasible due to their size, shape, and number (Figure 3) [7].

**Figure 4:**
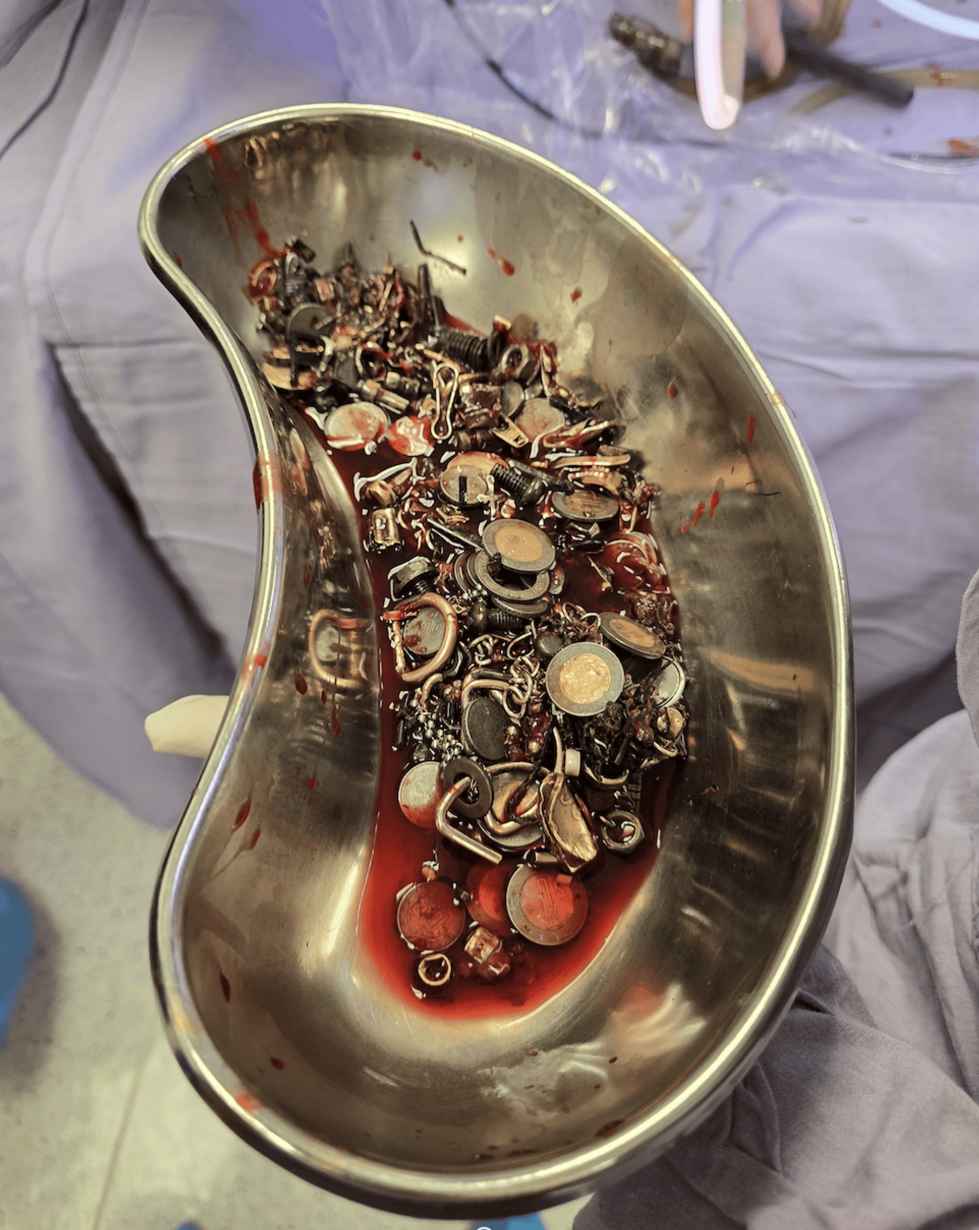
Metallic objects retrieved from the gastric chamber and duodenum through surgical extraction.

Surgical management was undertaken after failed minimally invasive attempts and concern for complications. Laparoscopic access was initially attempted but proved unsuccessful due to the bulk and irregularity of the material, prompting conversion to open surgery, which allowed complete removal of all metallic objects without intraoperative complications (Figure 4).

Given the patient’s poor nutritional status, postoperative care included one week of in-hospital monitoring to ensure surgical success and allow correction of altered biochemical parameters associated with malnutrition.

Following the surgical intervention, the patient was referred to the Psychiatry service of the same hospital to verify whether financial constraints were preventing access to treatment or if the patient was refusing medication. This evaluation supported a structured interdisciplinary approach, coordinating psychiatric care with ongoing surgical follow-up, ensuring both behavioral management and prevention of recurrence.

## Discussion

Bezoar classification and epidemiology

Phytobezoars are the most common, accounting for up to 70% of cases, formed primarily from undigested fibers or plant-based foods. A notable subtype, diospirobezoar, develops after the ingestion of persimmon (*Diospyros kaki*) [2-11]. Lactobezoars, composed of milk proteins, predominantly affect premature infants. Trichobezoars, associated with psychiatric disorders like trichotillomania, resist digestive enzymes. Rapunzel syndrome is a rare variant that extends from the stomach into the small intestine or colon [2-10]. Pharmacobezoars consist of drug conglomerates and are influenced by drug properties and gastrointestinal pH [8-17].

Metal bezoars are exceptionally rare, arising from ingestion of metallic objects such as coins, screws, nails, or nuts [11,12]. Plastic bezoars from materials like rubber bands or toys have also been reported. Gastric bezoars are uncommon (~0.5% of cases), while small intestine bezoars range from 0.4% to 4% [2-6].

Clinical significance

Metal bezoars can cause severe gastrointestinal obstruction and are most often seen in patients with psychiatric or compulsive ingestion behaviors. This case illustrates the diagnostic complexity and therapeutic challenges of these unusual foreign bodies. Due to their size, irregular shape, and complication risk, surgical removal is frequently necessary. Endoscopic extraction was not feasible, making open surgery the safest approach.

Multidisciplinary management

Metal bezoars often reflect underlying psychiatric or behavioral disorders, emphasizing the need for comprehensive evaluation to prevent recurrence. In this case, psychiatric assessment and attention to the patient’s history of polysubstance abuse were crucial, consistent with literature recommendations. Early and accurate diagnosis is vital, as bezoars - though rare - can lead to severe outcomes if not promptly addressed. Imaging modalities such as radiography and computed tomography are essential, and findings must be interpreted alongside a detailed clinical history of possible foreign body ingestion.

Treatment decisions should be individualized based on bezoar size, composition, and location, as well as the overall condition of the patient. Here, the metallic nature and potential complications necessitated surgery. This case underscores the importance of a multidisciplinary approach, integrating surgical intervention, psychiatric evaluation, and structured follow-up to achieve favorable long-term outcomes.

## Conclusions

This case highlights the rarity and complexity of metal bezoars as a cause of gastrointestinal obstruction, particularly in patients with a history of chronic substance abuse and compulsive behaviors. The inability to perform endoscopic removal due to the size and characteristics of the foreign bodies emphasized the necessity of open surgical intervention for safe and effective extraction. Furthermore, it underscores the importance of a multidisciplinary approach that includes not only surgical management but also psychiatric evaluation and treatment to address underlying factors and prevent recurrence. Early detection through imaging studies and a thorough clinical history is essential to improve outcomes in these uncommon yet potentially serious cases.
